# Laboratory-Acquired Dengue Virus Infection, United States, 2018

**DOI:** 10.3201/eid2607.191598

**Published:** 2020-07

**Authors:** Tyler M. Sharp, Teresa G. Fisher, Kristin Long, Garry Coulson, Freddy A. Medina, Carolyn Herzig, Mary Beth Koza, Jorge Muñoz-Jordán, Gabriela Paz-Bailey, Zack Moore, Carl Williams

**Affiliations:** US Public Health Service, Rockville, Maryland, USA (T.M. Sharp);; Centers for Disease Control and Prevention, San Juan, Puerto Rico, USA (T.M. Sharp, F.A. Medina, J. Jorge Muñoz-Jordán, G. Paz-Bailey);; North Carolina Department of Health and Human Services, Raleigh, North Carolina, USA (T.G. Fisher, K. Long, C. Herzig, Z. Moore, C. Williams);; University of North Carolina at Chapel Hill, Chapel Hill, North Carolina, USA (G. Coulson, M.B. Koza);; Centers for Disease Control and Prevention, Atlanta, Georgia, USA (C. Herzig)

**Keywords:** dengue, laboratory-acquired infection, biosafety, viruses, high-titer viruses, United States, dengue virus, North Carolina

## Abstract

Investigation of a dengue case in a laboratory worker in North Carolina, USA, revealed that the case-patient prepared high-titer dengue virus stocks soon before illness onset. Improper doffing of gloves with an open finger wound likely resulted in cutaneous exposure. This case reinforces recommendations for enhanced precautions when working with high-titer dengue virus.

Four genetically distinct but serologically related dengue viruses (DENV-1–4) cause dengue, an acute febrile illness common throughout the tropics (*1*). DENV is transmitted by *Aedes* mosquitoes and has a median incubation period of 6 days (*2*). Other routes of DENV transmission include perinatal (*3*), blood transfusion (*4*), needle stick (*5*), and laboratory exposure (*6*–*8*).

In August 2018, the North Carolina Department of Health and Human Services (NCDHHS; Raleigh, North Carolina, USA) was notified of a dengue case in a laboratory worker. NCDHHS and CDC conducted an investigation to identify the most likely route of exposure.

## The Study

**Figure 1:**
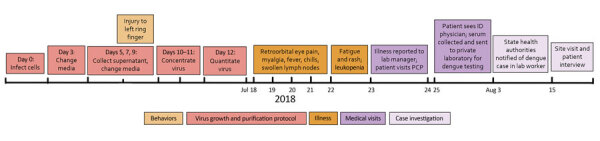
Timeline of events surrounding a case of laboratory-acquired dengue virus infection, United States, 2018. ID, infectious disease; PCP, primary care physician.

We interviewed the case-patient and reviewed medical records to collect travel history, potential exposures, clinical course, and diagnostic test results. The case-patient reported no recent travel to an area with ongoing DENV transmission. No travel-associated dengue cases were reported in 2018 from the county where the case-patient worked and resided. The case-patient reported illness onset on July 18, 2018, with retroorbital eye pain, fever, myalgia, arthralgia, lethargy, chills, and lymphadenopathy (Figure 1). By July 23, the case-patient was afebrile but had a whole-body maculopapular rash. The case-patient was evaluated by a physician that day and received a diagnosis of viral illness. After reporting the illness to the institutional occupational health clinic, the case-patient was referred to an infectious disease physician. Upon evaluation 2 days later, vital signs and laboratory values were unremarkable except for leukopenia (3.3 × 10^6^ cells/mm^3^).

Four serum specimens were forwarded to CDC for diagnostic testing: a baseline specimen collected ≈1.5 years before illness onset; an acute specimen collected 7 days after illness onset; an early-convalescent specimen collected ≈1 month after illness onset; and a late-convalescent specimen collected ≈6 months after illness onset (Table). The acute specimen tested positive at a commercial laboratory for detection of nonstructural protein 1 (NS1) antigen and DENV IgM and negative for *Ehrlichia* IgG. At CDC, reverse transcription PCR (*9*) performed on the acute specimen was negative; DENV IgM and IgG were detected (*10*,*11*) in acute and convalescent specimens. In the baseline specimen, neutralizing antibodies were detected for yellow fever virus but not DENV or West Nile virus. Comparison of DENV neutralizing antibody titers in acute, early convalescent, and late convalescent serum specimens confirmed incident DENV infection; however, a >4-fold rise in neutralizing antibody titer against multiple DENVs precluded identification of the specific infecting DENV.

**Table:** Summary of diagnostic test results of serum specimens collected from a case-patient with laboratory-acquired DENV infection, United States, 2018*

Specimen designation	Time of specimen collection†	rRT-PCR	NS1 ELISA	IgM ELISA	DENV IgG ELISA titer	Neutralizing antibody titer					
						DENV-1	DENV-2	DENV-3	DENV-4	WNV	YFV
Baseline‡	−1.5 y	NT	NT	NT	1:40	<20	<20	<20	<20	<20	40
Acute‡	7 d	Neg	Pos	Pos	1:163,840	<80	160	640	640	<80	160
Early convalescent‡	28 d	NT	NT	Pos	1:163,840	80	640	1280	320	40	80
Late convalescent§	190 d	NT	NT	Pos	1:40,960	40	<20	160	160	NT	NT

*DENV, dengue virus; IgM ELISA, anti-DENV IgM antibody capture enzyme linked immunosorbent assay; NS1 ELISA, nonstructural protein 1 ELISA; NT, not tested; rRT-PCR, real-time reverse transcription PCR; WNV, West Nile virus; YFV, yellow fever virus. †Relative to illness onset. ‡Neutralizing antibody titers obtained by 90% plaque reduction neutralization test. §Neutralizing antibody titers obtained by recombinant microfluorescence reduction neutralization test.

We visited the research laboratory where the case-patient worked; the principal investigator and laboratory safety officer described laboratory safety protocols. We reviewed laboratory activities performed by the case-patient in the week before illness onset and interviewed the case-patient regarding practices of donning and doffing personal protective equipment (PPE).

In the 2 weeks before illness onset, the case-patient reported working with a protocol to grow, purify, and concentrate DENV-4. The case-patient reported wearing a single pair of nitrile gloves, eye protection, a lab coat, and closed-toed shoes while working with infectious virus in a certified biosafety cabinet (BSC).

The protocol for virus production and concentration included inoculating ≈40 roller bottles of Vero cells with ≈10^6^ plaque-forming units (PFU) of DENV-4 (Figure 1). Media were harvested and pooled on days 5, 7, and 9 postinoculation and concentrated by tangential flow filtration followed by sucrose gradient fractionation. Fractions were collected by piercing the centrifugation tubes and collecting fractions using a safety mechanism that prevented needle sticks. Fractions were separated by sodium dodecyl sulfate polyacrylamide gel electrophoresis and protein concentration determined using a bicinchoninic acid assay. Typical protein concentrations correlated with virus titers of 10^9^–10^10^ PFU/mL. The case-patient also performed neutralization and ELISA assays for DENV-1–4 during the 2 weeks before illness onset.

The case-patient reported that small splashes often occurred during virus production and purification. The case-patient did not change gloves when splashes occurred but occasionally performed surface decontamination of gloves and the BSC with 70% ethanol. The case-patient estimated entering and exiting the BSC 6–8 times per day on most days of the protocol but not being vigilant about handwashing after removing gloves. The case-patient reported taking online Biosafety Level 2 (BSL-2) training upon joining the laboratory, receiving hands-on training for BSL-2 work, and annually reviewing laboratory safety plans and procedures.

**Figure 2:**
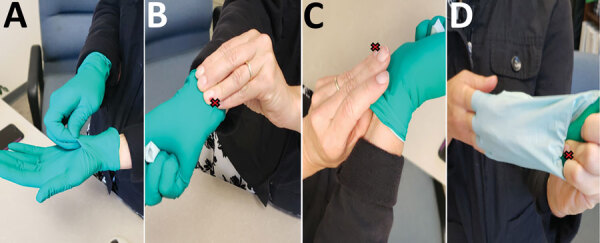
Depiction of improper protocol for doffing gloves the case-patient reported using while conducting a protocol for growth and purification of high-titer dengue virus, United States, 2018. The red X indicates the location of an open wound on the ring finger of the case-patient’s left hand.

The case-patient reported having sustained a compression wound on the ring finger of the left hand on July 9 or 10; the wound later appeared infected and oozing. The case-patient reported not bandaging or covering this wound before donning a single pair of gloves while working on the protocol for virus production and purification. The case-patient demonstrated their technique for doffing gloves (Figure 2): the base of the glove of the left hand was pinched with the thumb and forefinger of the right hand and the glove removed while turning it inside out, after which the base of the glove on the right hand was pinched with the thumb and forefinger of the now ungloved left hand. The case-patient acknowledged that the wound on the ring finger of the left hand could have contacted the potentially contaminated glove on the right hand. The case-patient also mentioned having potentially touched mucosal surfaces of the nose or mouth with the lab coat sleeve while working with infectious virus in the BSC.

## Conclusions

The presence of an open finger wound during work with high-titer DENV coupled with improper glove doffing suggests that laboratory-acquired infection by cutaneous exposure was the most likely route of DENV infection in this case. However, other routes of exposure, including mucosal, could not be ruled out. 

Three previous cases of laboratory-associated DENV infection have been reported. In Nigeria, a laboratorian responsible for cleaning cages and disposing of mice infected with DENV-1 became infected, although mosquito-borne transmission could not be ruled out (*6*). A laboratorian in Australia was infected while working with DENV-2 (*7*), although it could not be determined if infection occurred from a bite from an infected mosquito in the laboratory or potential mucocutaneous exposure while working with infectious virus. In South Korea, a laboratorian was infected with DENV-2 following a needle stick injury while filtering cell cultures of DENV-2 (*8*).

In this case, detection of NS1 antigen independently confirmed acute DENV infection, supported by detection of DENV IgM and >4-fold rise in DENV IgG and DENV neutralizing antibody. However, historic exposure to >1 flavivirus complicated interpretation of neutralizing antibody titers and precluded identification of the infecting DENV. Moreover, we could not rule out infection with DENV between collection of the baseline and acute specimens. The difficulty interpreting flavivirus neutralizing antibody patterns during secondary infections is well described (*12*).

A study in Belgium conducted during 2007–2012 found that only 40% of laboratory-associated infections occurred following a known exposure event; a definitive cause of exposure could not be identified in nearly one third of cases associated with bloodborne pathogens (*13*). Thus, laboratory-acquired infections, including those with DENV, likely occur more frequently than have been documented.

Titers of infectious DENV in human blood resulting from mosquito-borne transmission are typically 10^3^–10^7^ PFU/mL (*14*). At 10^9^–10^10^ PFU/mL, the concentration of DENV the case-patient handled would have been 100 to 10 million times higher than the concentration found in the average blood specimen of a patient with DENV infection. Although BSL-2 containment is recommended for laboratory work with DENV, enhanced safety precautions including double-gloving are recommended when handling large-scale or high-titer virus (*15*). This investigation highlights the importance of developing and maintaining risk assessment and management programs to mitigate exposures to infectious agents and emphasizing good microbiological practices and procedures training for laboratorians, including proper PPE donning and doffing techniques.
